# Foundational knowledge regarding childhood obesity: a cross-sectional study of medical students

**DOI:** 10.1186/s12889-019-7499-1

**Published:** 2019-09-11

**Authors:** Emily Hill Guseman, Elizabeth A. Beverly, Jonathon Whipps, Sophia Mort

**Affiliations:** 1 Diabetes Institute, Ohio University, Athens, OH 45701 USA; 2 Department of Family Medicine, Ohio University Heritage College of Osteopathic Medicine, Athens, OH 45701 USA; 3 Translational Biomedical Sciences, Ohio University, Athens, OH 45701 USA; 4 Department of Medicine, Ohio University Heritage College of Osteopathic Medicine, Athens, OH 45701 USA

**Keywords:** Weight management, Obesity screening, Medical education, Primary care

## Abstract

**Background:**

Documentation and diagnosis of childhood obesity in primary care is poor and providers are often unfamiliar with guidelines. This lack of knowledge may be attributed to insufficient training in medical school and residency; however, no studies have evaluated medical students’ knowledge of recommendations.

**Methods:**

We distributed a modified version of the *Physician Survey of Practice on Diet, Physical Activity, and Weight Control* to medical students at a single university. Descriptive analyses assessed knowledge and attitudes of childhood obesity and diabetes.

**Results:**

Of the 213 participating students, 74% indicated being unfamiliar with obesity screening recommendations. Few correctly identified BMI percentile cut-points for child overweight (21.2%), obesity (23.7%), and normal weight (29.4%). They reported screening glucose 4.5 years earlier in patients with risk factors compared to those without (*p* < 0.001).

**Conclusions:**

Although students recognized the need for earlier diabetes screening in children with risk factors, we determined that overall, student knowledge of obesity-related preventative care was inadequate.

**Electronic supplementary material:**

The online version of this article (10.1186/s12889-019-7499-1) contains supplementary material, which is available to authorized users.

## Background

Current data indicate that 18.5% of children (age 2–19 years) in the United States (US) have obesity [1]. Despite increased awareness, childhood obesity remains a public health crisis and efforts toward improvement have been unsuccessful. Children with obesity are at higher risk for obesity in adulthood [2] and experience adverse outcomes both physiologically [3] and psychologically [4, 5]. In 2007, the American Academy of Pediatrics published an Expert Committee report including new recommendations for the screening, treatment, and prevention of obesity [6], which were upheld by the US Preventative Services Task Force (USPSTF) in 2017 [7]. The report outlines a staged approach to pediatric obesity management that emphasizes the importance of the interactive relationships not only between the at-risk child and his/her family, but also the vital role of the primary care provider (e.g., pediatrician, family physician, family nurse practitioner) to broadly reduce the risk of excess weight gain.

The staged treatment model proposed by the Expert Committee consists of four stages, gradually increasing in intensity and complexity. Stage 1 – also referred to as “prevention plus” – focuses on assessment, preventative health messages, and early intervention at the level of the primary care provider’s office. Through this model, the primary care provider gives anticipatory guidance to *all* patients and families. All children and families, regardless of the child’s weight status, should receive education regarding healthy dietary behaviors, physical activity, and patterns of sedentary behaviors. Stages 2 through 4 feature progressively increasing treatment intensity that is initiated when the provider determines more directed weight management is necessary. In stage 2, the Expert Committee recommends the addition of planned and structured meals, snacks, and physical activity, and the addition of specific goal-setting and the use of logs and other reinforcement tools. In stage 3, parents and children work together with a multidisciplinary team, often at specialty weight management clinics, and treatment includes a focus on obesity-related comorbidities. Finally, in stage 4, treatment may include intensive interventions such as very low-calorie diets and in extreme cases, metabolic surgery [6].

Treatment of obesity necessitates an understanding of the bidirectional relationship between a patient’s weight status (i.e., obesity) and psychological well-being (i.e., feelings of value and respect) and a focus not on weight but on promoting and supporting healthy behaviors. Best practices for assessment of obesity, which are reflected in the Expert Committee’s report, include accurate anthropometry using calibrated equipment, calculation of body mass index (BMI) percentile for age and sex, assessment of family and personal history, assessment of diet and physical activity-related behaviors, and assessment of readiness to change [8].

In the decade since these Expert Committee guidelines were released, a number of research teams have evaluated adherence at the level of the primary care provider’s office. Studies generally indicate that adherence to documentation recommendations is poor and providers are often unfamiliar with the guidelines [9–15]. Providers’ lack of knowledge regarding the Expert Committee guidelines may be attributed to insufficient training in medical school and residency [16]; however, no studies have evaluated medical student knowledge of these recommendations or the extent to which they are included in the curriculum. Thus, the purpose of this study was to evaluate osteopathic medical students’ knowledge of the Expert Committee recommendations for screening and prevention of overweight and obesity, and whether improvement is seen as students progress through the undergraduate medical curriculum.

## Methods

This study was cross-sectional in nature. An adapted version of the National Cancer Institute’s (NCI) *Physician Survey of Practices on Diet, Physical Activity, and Weight Control: Questionnaire on Child/Adolescent Care* [17] was distributed electronically to first- through fourth-year medical students; modifications to the questionnaire are described below. The research team distributed the questionnaire via email in November 2017; a reminder email with the questionnaire was distributed 3 weeks later. The Ohio University Office of Research Compliance approved the protocol and all recruitment procedures and materials.

### Subjects

Medical students from three campuses (Athens, Cleveland, Dublin) and clinical practice partners associated with the Ohio University Heritage College of Osteopathic Medicine were invited to participate in an electronic, anonymous survey assessment of pediatric obesity-related knowledge, beliefs, and behaviors. Participation in the study was completely voluntary. In return for their participation, students were entered into a drawing for one of eight $100.00 gift cards by clicking on a new Qualitrics link, thus preventing linking of responses to student names or email addresses.

### Measures

The questionnaire used for this study was a modified version of the NCI questionnaire entitled *Physician Survey of Practices on Diet, Physical Activity, and Weight Control* [17]. Questions about sociodemographic factors (age, sex, race/ethnicity), likelihood of specializing in primary care and pediatrics, and estimated number of hours in specific clinical care settings were added to the beginning of the questionnaire. Questions from the NCI questionnaire were modified slightly to apply to medical students. For instance, the original question “How often do you assess the following in children or adolescents (ages 2-17)?” was changed to “How often do you think physicians should assess _____ in children or adolescents (ages 2-17 years)?”. The full survey used for this study is included as an Additional file 1. The questions were a combination of Likert scale (quantitative) responses and open-ended (qualitative) responses.

### Data collection

Students completed the questionnaire online via the online service Qualtrics (Provo, UT: Qualtrics). Qualtrics permitted our research team to download participants’ questionnaire responses into a spreadsheet without including identifying information (i.e., email address, name) to protect their confidentiality. Students were recruited via an email distribution list sent to current first- through fourth-year students associated with Ohio University Heritage College of Osteopathic Medicine. The email included a brief introduction to the study and a web link that directed them to the questionnaire. All participants provided informed consent prior to participation.

### Data analysis

Basic sociodemographic characteristics were assessed using frequencies and descriptive characteristics and are presented as means, standard deviations, and sample percentages. Frequencies of individual question responses were also calculated and presented as percentages. Multinomial logistic regression was used to evaluate whether clinical stage predicted familiarity with the Expert Committee guidelines, combining “very familiar” and “somewhat familiar” due to low response frequency that would preclude statistical analysis. Student responses to questions regarding body mass index (BMI) cut-points for overweight (85th–94.9th percentile), obesity (≥ 95th percentile), and normal weight (5th–84.9th percentile) were coded as correct or incorrect and knowledge of cut-points was evaluated by response frequency. Responses to questions regarding current physical activity recommendations for children [18] were coded as correct or incorrect by two of the researchers (EHG and JW); logistic regression was used to evaluate whether clinical stage predicted correct answers to questions about physical activity recommendations and screening guidelines. Independent t-tests were used to evaluate whether hours working in pediatric primary care differed according to response (correct vs. incorrect). Finally, we used paired t-tests to evaluate whether the age at which students would first order glucose screening differed by child risk factor status. Statistical analyses were performed using SPSS v. 24.0 and significance was accepted at *p* < 0.05.

## Results

Of the 926 students emailed, a total of 238 consented to participate (25.7% return rate). Twenty-five students were removed from the analysis due to survey non-completion: 14 students completed only demographic characteristics and an additional 11 students did not answer any of the survey questions included in this analysis. Thus, the final sample included 213 students whose characteristics are displayed in Table 1. Twenty-four percent of the sample was in their first year (*n* = 52), 38.0% in the second year (*n* = 81), 19.2% (*n* = 41) in the third year and 18.3% (*n* = 39) in the fourth year. Thirty-eight percent of respondents (*n* = 81) met physical activity recommendations themselves, most (59%, *n* = 125) were within the healthy range for BMI, and only 15% (*n* = 31) met both fruit and vegetable intake recommendations.

**Table 1:** Demographic characteristics of the sample

	Total (*n* = 213)	Pre-clinical (*n* = 133)	Clinical (*n* = 80)
Gender			
Female	122 (57.3%)	76 (57.1%)	46 (57.5%)
Male	91 (42.7%)	57 (42.9%)	34 (42.5%)
Race			
Asian	13 (6.1%)	8 (6.0%)	5 (6.3%)
Native Hawaiian/Pacific Islander	2 (0.9%)	1 (0.8%)	1 (1.3%)
White	169 (79.3%)	101 (75.9%)	68 (85.0%)
African American	14 (6.6%)	9 (6.8%)	5 (6.3%)
Other	15 (7.0%)	14 (10.5%)	1 (1.3%)
Ethnicity			
Hispanic/Latino	4 (1.9%)	3 (2.3%)	1 (1.3%)
	Mean (SD)	Mean (SD)	Mean (SD)
Likelihood of specializing in …			
Primary Care (%)	55.6 (30.8)	56.0 (27.4)	54.8 (36.7)
Pediatrics (%)	33.3 (30.0)	35.5 (28.9)	27.8 (32.2)
Experience interacting with patients in …			
Primary care (h)	307.1 (649.1)	191.0 (671.1)	509.0 (557.6)*
Pediatrics (h)	61.2 (138.3)	21.4 (62.1)	129.7 (195.8)*

* Denotes statistically significant difference (*p* < 0.05)

The majority of students were “somewhat unfamiliar” or “very unfamiliar” with the Expert Committee guidelines for screening and treatment of childhood and adolescent obesity. Multinomial logistic regression indicated that, compared to students in the third and fourth years of medical study, preclinical students (years 1 and 2) were significantly less likely to choose “familiar” (“very familiar” [*n* = 3] and “somewhat familiar” [*n* = 41] combined due to low n; OR = 0.231, *p* < 0.001) or “somewhat unfamiliar” (OR = 0.63, *p* = 0.166) than “very unfamiliar”. Students were generally unable to correctly identify BMI percentile cut-points for overweight (70.9%; *n* = 151 incorrect) and obesity (68.1%; *n* = 145 incorrect); an additional 11 students (5.2%) did not answer these questions. Clinical stage did not predict a correct answer to either of these questions (*p* = 0.44 and *p* = 0.20, respectively). Students correctly identified BMI percentiles for normal weight more frequently (33.3%; *n* = 71), though 47 students (22.1%) did not answer this question. Hours in pediatric primary care did not differ between students who selected the correct BMI percentile and those who did not.

The majority of students were unfamiliar with current physical activity recommendations for children. Slightly less than one-quarter of the sample (22.5%, *n* = 48) correctly identified *both* recommended daily minutes and days per week (60 min per day, 7 days per week). Students were more successful in identifying the recommendation for 3 days per week of vigorous physical activity. (39.9%, *n* = 85) and muscle- and bone-strengthening activity (42.7%, *n* = 91). Again, logistic regressions evaluating whether clinical stage predicted correct answers were non-significant (*p* = 0.23 to *p* = 0.74). Most students indicated that they would seek additional information regarding physical activity recommendations from professional organizations, including physician organizations (e.g., American Academy of Pediatrics, American Academy of Family Physicians, etc.) and public health entities (e.g., Centers for Disease Control and Prevention, World Health Organization), followed by journals and clinical resources (e.g., UpToDate, Clinical Key). Relatively few students indicated that they would seek information from other websites (e.g., Google, Reddit) or other professionals (e.g., registered dietitians, professors) (Table 2). Notably, no student indicated that they would consult with an exercise professional (e.g., exercise physiologist, exercise specialist, physical therapist).

**Table 2:** Categorized student responses to the question, “Where would you look to find more information regarding current physical activity recommendations for children/adolescents (2-17 years)?”

	Frequency n (%)
Journals/clinical resources	26 (13.6)
Physician organizations	79 (41.4)
Public health/government organizations	62 (32.5)
Other websites	22 (11.5)
Other professionals	2 (1.0)

**Fig. 1:**
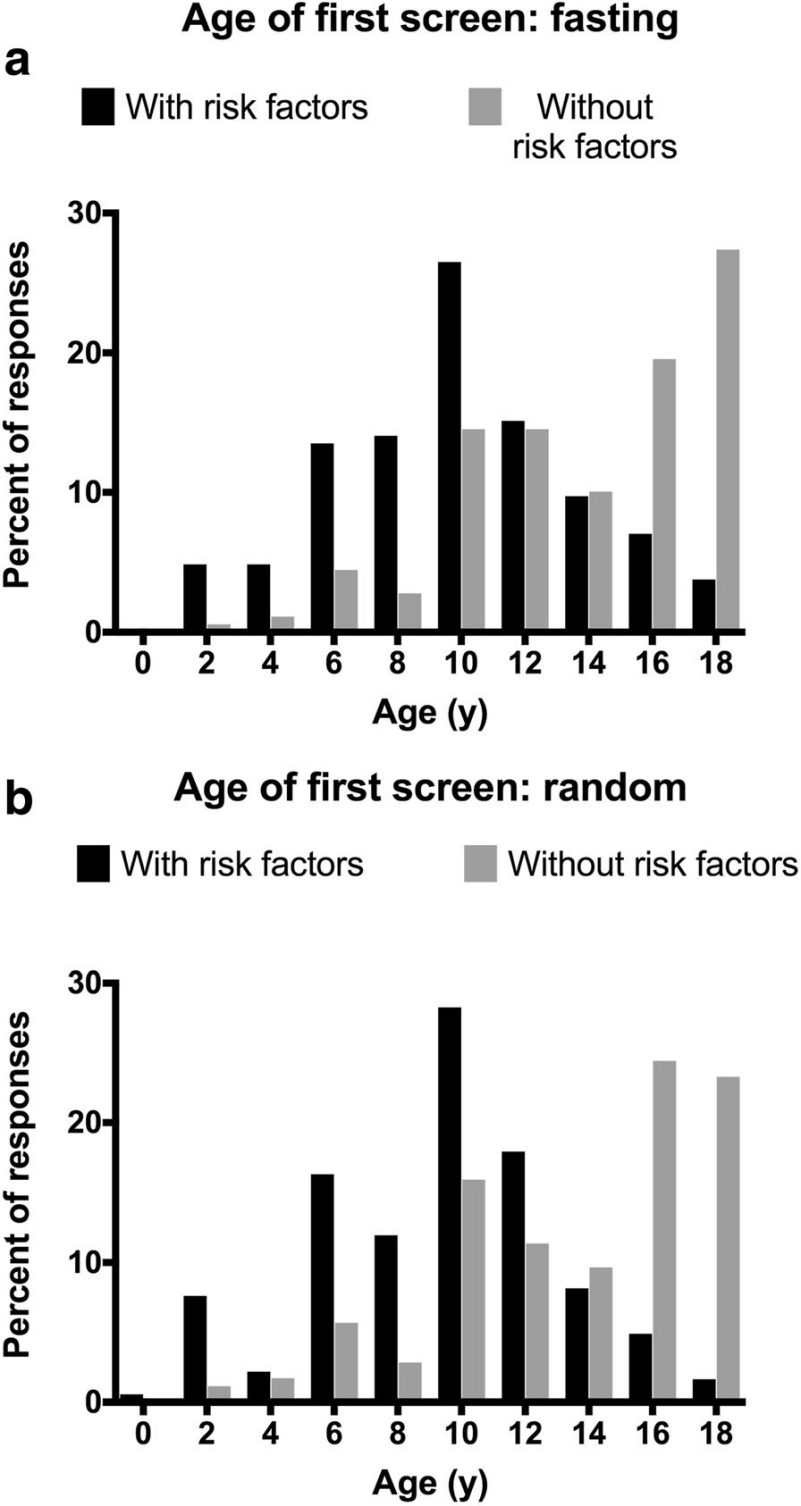
**a** Age of first fasting blood glucose screening, with risk factors compared to without risk factors. (means significantly different, *p* < 0.001). **b** Age of first random blood glucose screen, with risk factors vs. without. (means significantly different, *p* < 0.001)

Next, participants were asked questions regarding the age at which they would first recommend fasting or random glucose screening for children with and without risk factors for type 2 diabetes. Overall, students indicated that they would screen children with risk factors on average about 4 years earlier than those without (*p* < 0.001, see Fig. 1; random mean age = 9.2 ± 4.0 years with risk factors vs. 13.6 ± 4.6 years without; fasting mean age = 9.8 ± 4.4 years with risk factors vs. 14.2 ± 5.2 years without). Students most commonly indicated an age at initiation of glucose screening of 10 years for three of the four scenarios: random screening with risk factors, random screening without risk factors, and fasting screening with risk factors. For fasting screening without risk factors, the mode was 17 years.

## Discussion

Our study presents several findings that suggest medical students need additional knowledge to provide effective care for obesity in childhood. The majority of medical students surveyed indicated being unfamiliar with Expert Committee guidelines for screening and treatment of childhood and adolescent obesity. Students in the first and second year were less likely to perceive themselves as familiar with the Expert Committee recommendations as compared to third- and fourth- year students. This may indicate that students do encounter these recommendations to some degree on rotations, but that this experience is minimal or limited and insufficient to support complete knowledge of recommendations. Further, the majority of respondents were unfamiliar with current physical activity recommendations for children; this finding did not differ by pre-clinical or clinical stage in medical school. Notably, students reported that they would seek information about physical activity recommendations from professional organizations rather than consult with exercise professionals. Finally, students recommended fasting or random glucose screening for children with risk factors for type 2 diabetes an average of 4 years earlier than for children without risk factors.

Our findings are consistent with previous studies showing that primary care physicians [12, 13] and nurse practitioners [11] are generally unfamiliar with recommended practices for obesity screening and treatment. Despite the original release of the Expert Committee recommendations in 2007 [6] and the recent USPSTF report upholding the recommendations [7], physician knowledge of the recommendations and practices related to obesity prevention and treatment do not show evidence of improvement [9, 15]. Low usage of recommended BMI percentile cut-points for childhood overweight and obesity by physicians [12] may partially explain why so few respondents correctly identified cut-points and why this did not differ between pre-clinical and clinical stage students or according to clinical experience hours in pediatric primary care. Further, most students incorrectly identified current physical activity guidelines for children. These results suggest that medical students are not learning obesity-specific content knowledge in pre-clinical coursework or in their clinical rotations, despite perceiving themselves to be more familiar with recommendations in the latter stages of training.

The past decade has also featured changes to recommendations regarding early screening of blood glucose among children and adolescents at risk for type 2 diabetes and other cardiometabolic complications. Current guidance from the American Diabetes Association suggests that children and adolescents should undergo type 2 diabetes screening every 3 years, beginning at the onset of puberty or 10 years of age, in cases of overweight and obesity with at least one additional risk factor [19, 20]. Students responding to our survey generally indicated that they would initiate glucose screening approximately 4.5 years earlier in overweight/obese patients with risk factors than those with perceived lower risk (*p* < 0.001). This was true both for random glucose screening and fasting glucose screening, which is consistent with current professional guidelines to screen earlier in the presence of risk factors. Students most commonly indicated an age at initiation of glucose screening of 10 years for three of the four scenarios; only fasting screening without risk factors differed (17 years). Notably, there was a large degree of variation in student responses to these questions as shown by the wide standard deviations. Further, a number of students indicated they would not begin glucose screening until adulthood (*n* = 1 to *n* = 9, depending on scenario). To our knowledge, age at onset of glucose screening has not been evaluated in any previous studies in the United States; as such, we are unable to compare our findings to those of others.

We have previously shown that medical students believe physical activity and lifestyle counseling to be both part of a primary care physician’s job and important to incorporate in preventative medical care [21]. These findings are encouraging and suggest that students are aware of the importance of early screening and lifestyle counseling among children and adolescents at risk for developing type 2 diabetes. However, our results suggest that students may not accurately identify those children who are at increased risk, as demonstrated by relatively poor identification of BMI percentile cut-points. Thus, it is important to provide medical students with appropriate training in physical activity and other lifestyle recommendations (e.g., dietary guidelines, sleep habits, sedentary patterns) and encourage integration of these counseling practices early, and frequently, in the course of medical education. Unfortunately, limited research is available regarding a curricular focus on obesity during medical school, particularly regarding childhood-specific training.

A 2012 systematic review focusing on educational interventions for obesity and obesity related behaviors included only five studies with sufficient outcomes for review [22]. Most studies were superficial in nature, focused primarily on assessment of anthropometric characteristics, and ineffective at improving students’ attitudes toward obesity treatment. While the content-heavy nature of pre-clinical education leaves little time for inclusion of additional material, data do not suggest that physicians receive sufficient pediatric obesity training on rotations or in residency, either. Most residents participating in one study reported having received training in obesity and obesity diagnosis. Further, most felt that the amount of training they received was appropriate and believed they were able to provide effective counseling [23]. However, a study of primary care residents found that less than half actually answered obesity-related knowledge questions correctly on a survey about lifestyle counseling [24]. In another study, pediatric residents were more likely to identify overweight or obese patients and refer them to appropriate follow-up services than attendings or nurse practitioners; however, over half of the patients who were overweight or obese were still not diagnosed appropriately [25]. Thus, although some providers feel prepared to counsel patients, a lack of knowledge persists at all levels of care, including primary care physicians, residents, and nurse practitioners [9–12, 24, 25].

Study limitations include homogeneity of the study sample from one medical school with three campuses in a Midwestern state, the cross-sectional study design, the modest response rate, and respondents’ self-reported data. Data from one school limits the ability to generalize the findings to other medical schools. However, it should be noted that the three campuses for this single medical school reside in very different geographical regions across the state. Next, only 25.7% of the medical students who were invited to participate in the study had complete data; 25 cases were removed from the analysis due to missing data which led to a 3% decrease in response rate. This is true despite incentivizing study participation, which may indicate that simply being entered into a drawing for one of eight gift cards was not sufficient incentive. As such, the students who volunteered may have been more willing or motivated to answer questions about pediatric obesity screening and treatment compared to the students who did not participate. For these reasons, the self-reported findings are susceptible to selection bias. Our modifications to the questionnaire may have also influenced the likelihood of bias. Future research with a larger, more heterogeneous sample should include students from multiple medical schools. Further, additional training in childhood obesity screening and treatment across all 4 years of medical school is needed to increase familiarity with screening guidelines, appropriate assessment, and physical activity recommendations for children.

## Conclusions

Our findings indicate that medical students are generally unfamiliar with 2007 Expert Committee recommendations for the prevention and treatment of obesity in childhood and unable to identify age-appropriate cut-points for weight status and physical activity. Students did recognize the need to implement diabetes screening at an earlier age for children with type 2 diabetes risk factors, though many students would initiate this screening beyond the onset of puberty or 10 years of age. Taken together with studies demonstrating similarly low knowledge among residents, pediatricians, and other primary care providers, there is a clear need to emphasize systematic approaches to obesity and diabetes risk reduction in medical school and ongoing medical education.

## Additional file

Additional file 1: OMS stage 1 questionnaire IRB. (DOCX 82 kb)

## Data Availability

The datasets used and/or analyzed during the current study are available from the corresponding author (gusemane@ohio.edu) on reasonable request.

## Abbreviations

BMI: Body mass index
NCI: National Cancer Institute
USPSTF: US Preventative Services Task Force
