# Supplementary material for: Foundational knowledge regarding childhood obesity: a cross-sectional study of medical students
Source: BMC Public Health. 2019 Sep 11;19:1251. doi: 10.1186/s12889-019-7499-1 (PMC6737597; doi:10.1186/s12889-019-7499-1)
Supplement: Supplementary file 1 — OMS stage 1 questionnaire IRB. (DOCX 82 kb) [file 12889_2019_7499_MOESM1_ESM.docx]

Demographic

What is your current year in medical school?

Which of the following best represents your gender identity?

Female

Male

Other

What do you consider to be your race?

American Indian or Alaska Native

Asian

Black or African-American

Native Hawaiian or Other Pacific Islander

White

Do you consider yourself to be Hispanic or Latina/o?

Yes

No

On a scale of 1-100, how likely are you to specialize in Primary Care?

On a scale of 1-100, how likely are you to specialize in Pediatrics?

Please estimate the number of hours you have spent interacting with patients in a *Primary Care* setting

Please estimate the number of hours you have spent interacting with *pediatric* patients in a *Primary Care* setting

Please estimate the number of hour you have spent interacting with patients in a clinical setting that is *not* primary care

Personal Beliefs

Please indicate how strongly you agree with each of the following statements:

1. Physicians have a responsibility to promote the following with their patients:
   1. Eat a healthy diet
   2. Be adequately physically active
   3. Maintain a healthy weight or lose weight
2. Patients are more likely to adopt healthier lifestyles if physicians counsel them to do so
   1. Strongly agree
   2. Somewhat agree
   3. Neither agree nor disagree
   4. Somewhat disagree
   5. Strongly disagree
3. There are effective strategies and/or tools to help patients:
   1. Eat a healthy diet
   2. Be adequately physically active
   3. Maintain a healthy weight or lose weight
4. I am confident in my ability to counsel my patients to:
   1. Eat a healthy diet
   2. Be adequately physically active
   3. Maintain a healthy weight or lose weight
5. I am effective at helping my patients:
   1. Eat a healthy diet
   2. Be adequately physically active
   3. Maintain a healthy weight or lose weight
6. To effectively encourage patient adherence to a healthy lifestyle, a physician must adhere to one him/herself.
   1. Strongly agree
   2. Somewhat agree
   3. Neither agree nor disagree
   4. Somewhat disagree
   5. Strongly disagree
7. Specifically, a physician will be able to provide more credible and effective counseling if he/she:
   1. Eats a healthy diet
   2. Is adequately physically active
   3. Maintains a healthy weight or loses weight

Are you familiar with the AAP guidelines for screening and treatment of childhood and adolescent overweight?

1. Very familiar
2. Somewhat familiar
3. Somewhat unfamiliar
4. Very unfamiliar

How often do you think physicians should assess weight (measured in office) in children or adolescents (age 2-17 years)?

Every well-patient visit

Every visit

Annually

As clinically indicated

Never

Other interval (specify)

How often do you think physicians should assess height (measured in office) in children or adolescents (age 2-17 years)?

Every well-patient visit

Every visit

Annually

As clinically indicated

Never

Other interval (specify)

How often do you think physicians should assess body mass index (BMI) in children or adolescents (age 2-17 years)?

Every well-patient visit

Every visit

Annually

As clinically indicated

Never

Other interval (specify)

How often do you think physicians should assess waist circumference or waist-to-hip ratio in children or adolescents (age 2-17 years)?

Every well-patient visit

Every visit

Annually

As clinically indicated

Never

Other interval (specify)

How often do you think physicians should assess weight for age growth charts in children or adolescents (age 2-17 years)?

Every well-patient visit

Every visit

Annually

As clinically indicated

Never

Other interval (specify)

How often do you think physicians should assess stature for age growth charts in children or adolescents (age 2-17 years)?

Every well-patient visit

Every visit

Annually

As clinically indicated

Never

Other interval (specify)

How often do you think physicians should assess BMI for age growth charts in children or adolescents (age 2-17 years)?

Every well-patient visit

Every visit

Annually

As clinically indicated

Never

Other interval (specify)

According to current guidelines, at what BMI percentile are child/adolescent patients (ages 2-17) considered to be...

1. Overweight
2. Obese

According to current guidelines, in what BMI percentile range are children or adolescents (ages 2-17) considered to have healthy weight? (check one box)

5^th^-65^th^ percentile

5^th^-75^th^ percentile

5^th^-85^th^ percentile

5^th^-95^th^ percentile

Other (specify)

Don’t know

For overweight/obese child/adolescent patients (age 2-17 years), at what age would you begin performing the following tests?

Random blood glucose testing for patients with risk factors or family history

Random blood glucose testing for patients without risk factors or family history

Fasting blood glucose testing for patients with risk factors or family history

Fasting blood glucose testing for patients without risk factors or family history

If you were providing routine care to pediatric patients **today**, what do you think would be the top 3 barriers to evaluating and/or managing your patients’ diet/nutrition, physical activity, and weight in your practice?

Not enough time

Not part of my role

I am not adequately trained in this area

Too difficult to evaluate and manage

Inadequate reimbursement

Lack of adequate referral services for diet, physical activity, and weight

Fear of offending the patient

Too difficult for patients to change their behavior

Lack of effective tools and information to give to patients

Lack of effective treatment options

Other (specify)

According to current guidelines, for children/adolescents (2-17 years), how much moderate physical activity is recommended for general health and prevention of chronic disease?

_____ minutes _____ days per week

According to current guidelines, how often should children/adolescents (2-17 years) participate in vigorous physical activities?

_____ days per week

According to current guidelines, how often should children/adolescents (2-17 years) participate in muscle and bone-strengthening activities?

_____ days per week

Where would you look to find more information regarding current physical activity recommendations for children/adolescents (2-17 years)? (free text field)

Personal Health Status/Health Behaviors

In general, would you say your health is:

Excellent

Very good

Good

Fair

Poor

These questions are about the foods you ate or drank during the past month, that is, in the past 30 days. Please include meals and snacks eaten at home, at work or school, in restaurants, and any place else. (choices – never, 1-3 times last month, 1-2 times per week, 3-4 times per week, 5-6 times per week, 1 time per day, 2 times per day, 3 or more times per day, 4 or more times per day, 5 or more times per day)

1. How often do you drink 100% fruit juice, such as orange, mango, apple, or grape juices? Do NOT include fruit drinks.
2. How often did you eat frit? Include fresh, frozen, or canned fruit. Do NOT include juices.
3. How often did you eat French fries or home fries or hash brown potatoes?
4. How often did you eat other potatoes? Include baked, boiled, mashed, or potato salad.
5. Not including potatoes (and not counting rice), how often did you eat other vegetables?

Moderate physical activities make you breathe somewhat harder than normal. During the last 7 days, did you do any moderate physical activities for at least 10 minutes? Think about activities such as bicycling, swimming, brisk walking, dancing, or gardening?

No (program to skip to vigorous)

Yes

On how many of the past 7 days did you do moderate physical activities? (blank days)

In the past 7 days, on a typical day in which you did moderate physical activities, how much time did you spend doing them? (blank minutes per day)

Vigorous activities make you breathe much harder than normal. Now think about vigorous activities you did that take hard physical effort, such as aerobics, running, soccer, fast bicycling, or fast swimming. During the last 7 days, did you do any vigorous physical activities in your free time for at least 10 minutes?

No (program to skip to strength)

Yes

On how many of the past 7 days did you do vigorous physical activities? (blank days)

In the past 7 days, on a typical day in which you did vigorous physical activities, how much time did you spend doing them? (blank minutes per day)

Now think about activities specifically designed to strengthen your muscles, such as lifting weights or other strength-building exercises. Include all activities even if you have included them before. During the last 7 days, did you do activities to strengthen your muscles?

No (program to skip to height and weight status)

Yes

How tall are you without shoes?

__ feet ___ inches

How much do you weight without shoes

______ pounds

If you are currently pregnant, how much did you weigh before your pregnancy?

______ pounds

Are you currently trying to: (check one)

Lose weight

Gain weight

Maintain weight

Not trying to make a change
